# Women’s mental health in Saudi Arabia: gender gaps, barriers to care, and a culturally integrated framework

**DOI:** 10.3389/fpsyg.2026.1943335

**Published:** 2026-10-22

**Authors:** Adeeb Obaid Alsuhaymi, Fouad Ahmed Atallah

**Affiliations:** Department of Sharia, College of Sharia and Law, Jouf University, Sakaka, Saudi Arabia

**Keywords:** anxiety, depression, SDG 3, mental-health stigma, postpartum depression, Saudi Arabia, somatization, women’s mental health

## Abstract

Women’s mental health remains underrepresented in empirical research and policy across the Gulf Cooperation Council (GCC), despite expanding national health-surveillance capacity. This study examines gender disparities in anxiety and depression among Saudi women, barriers to mental-healthcare access, and principles for culturally integrated care. A multi-source secondary analytical design integrated published aggregate indicators from four official Saudi national statistical sources with a structured, theory-informed synthesis of peer-reviewed evidence. The national-data component involved structured extraction, cross-source comparison, and integrated interpretation of published aggregate indicators; no individual-level microdata were re-analyzed and no pooled prevalence estimates were generated. The complementary literature component involved structured searching and abstract-level screening followed by purposive thematic selection from retrievable evidence to contextualize patterns identified in the national data rather than constitute a completed systematic review. The two evidence streams were integrated using the proposed Islamic Biopsychosocial–Spiritual (BPSS-I) framework, a theory-generating extension of Engel’s model that incorporates family-system dynamics, honor-based stigma, gender-specific determinants, and Islamic psychospiritual constructs. National indicators show severe anxiety in 9.0% of women compared with 6.9% of men. A substantial treatment gap is also evident: only 13.6% of Saudis with a lifetime mental disorder ever seek treatment, while approximately 80% of those with severe disorders receive no care. Reported postpartum depression prevalence varies considerably across Saudi settings and screening thresholds (approximately 15%–76%), with limited family and spousal support repeatedly identified as important psychosocial correlates. Broader evidence suggests that somatization may contribute to underrecognition of psychological distress, while stigma and family-level reputation concerns may shape help-seeking. Religiosity may function differently depending on positive or negative religious coping. Although women’s workforce participation has increased substantially under Vision 2030, available cross-sectional evidence does not establish corresponding changes in psychological distress or a causal relationship between reform and mental-health outcomes. The integrated analysis highlights the need for longitudinal Saudi-specific research, empirical validation of BPSS-I, and culturally adapted and spiritually responsive interventions tested within Saudi clinical and community settings.

## 1 Introduction

Mental disorders are among the leading contributors to the global burden of disease; depression and anxiety together affect more than 700 million people worldwide (World Health Organization, 2022). Women bear a disproportionate share: across world regions and cultural settings, female prevalence of depression and anxiety consistently exceeds male prevalence, with women roughly 1.6 times as likely as men to experience depression and panic disorders (Gater et al., 1998). This female excess is not biologically fixed but is substantially shaped by structural gender inequality—cross-national analysis across 122 countries shows that the female-to-male ratio in depressive-disorder rates tracks the Gender Inequality Index, independent of national income (Yu, 2018).

Within this global pattern, the Arabian Gulf presents a paradox. Gulf Cooperation Council (GCC) countries have invested substantially in women’s education and healthcare infrastructure, yet women’s mental health remains underserved and underdetected -and the gender gap is among the widest in the region. A GCC meta-analysis of 18,717 adults across 40 studies found depression prevalence to be broadly stable over two decades and statistically similar across the six GCC states, with young and female individuals consistently emerging as higher-risk groups; in the Saudi subsample, roughly one-fifth of participants showed moderate and about 8% severe depressive symptoms (Camia and Alhallami, 2023). Against this backdrop, the Saudi National Mental Health Survey (SNMHS), the Kingdom’s first nationally representative psychiatric-epidemiology study, found that 34% of Saudis met criteria for a mental disorder at some point in life, yet only 13.6% ever sought treatment and about 80% of those with severe disorders received no care (Altwaijri et al., 2019).

The burden among Saudi women is both acute and easily rendered invisible. National surveillance shows severe anxiety in 9.0% of women versus 6.9% of men, and moderate depression in 0.2% of women versus 0.1% of men (General Authority for Statistics, 2025a). Because these figures derive from self-report instruments in a high-stigma setting, they likely understate the true burden. Somatization—the expression of psychological distress through physical complaints—accounts for a large share of psychiatric presentations in Arab primary care (approximately 48% in an early United Arab Emirates [UAE] study; El-Rufaie et al., 1999) and has been documented in Saudi primary care as well (Alqahtani and Salmon, 2008), routing women through gastroenterology, orthopedics, and general medicine rather than mental-health services. In Riyadh primary care, physicians identified somatized depression at only slight agreement beyond chance (kappa = 0.27), well below accepted clinical thresholds (Becker et al., 2004).

These patterns call for a theoretical lens able to hold biological, psychological, and social factors together. Engel’s (1977) biopsychosocial (BPS) model—recently revitalized by Bolton (2023)—provides one: it holds that health and illness arise from the interaction of biological, psychological, and social systems rather than from disease biology alone, and it remains the dominant organizing framework in psychiatric epidemiology. Its application to non-Western, collectivist, and religiously grounded settings, however, has been criticized for underdeveloping the social domain and treating culture as ambient background rather than a causally active system (Hatala, 2012; Benning, 2015). Although the present study focuses specifically on Saudi women, evidence from broader Arab Muslim populations is relevant at this conceptual stage because Saudi-specific research on some culturally embedded mechanisms remains limited. Such evidence is used here to contextualize potentially relevant mechanisms rather than to imply cultural equivalence across Arab populations. Across this broader literature, extended family systems may simultaneously provide support and shape access to professional help (Fakhr El-Islam, 2008); honor-based stigma may make mental-health disclosure a concern for collective as well as individual reputation (Scull et al., 2014); reproductive transitions—particularly the postpartum period—highlight the importance of familial support as a modifiable correlate of depression outcomes (Zarroug et al., 2025); and spiritual beliefs may influence how distress is understood and whether professional help-seeking is perceived as appropriate or shameful (Fekih-Romdhane et al., 2023). These broader findings provide a comparative conceptual context; their relevance to Saudi women is examined against Saudi-specific evidence in the analysis that follows.

Narrowing the focus again to Saudi Arabia, the social context in which these determinants operate has also been changing under Saudi Vision 2030, the Kingdom’s national transformation program launched in 2016. Vision 2030 and associated reforms have introduced structural changes of direct relevance to women’s social and economic participation, including an increase in female labor-force participation from about 19% in 2017 to over 35% by 2022, changes to aspects of the male-guardianship system, and expanded women’s mobility, including the lifting of the ban on women driving in 2018 (Alghamdi et al., 2026). The gender-roles hypothesis—supported by WHO World Mental Health Survey data across multiple countries—suggests that changes in gender roles may, over time, be associated with a narrowing of the female excess in depression (Gater et al., 1998). Available cross-sectional evidence, however, does not establish whether Saudi Arabia’s recent social and economic changes have produced corresponding changes in women’s psychological wellbeing. Saudi women report general life satisfaction alongside substantial psychological distress, and about half of working women surveyed in Riyadh screened positive for mild-to-moderate depression and anxiety (Almadani and Alwesmi, 2023; Alateeq et al., 2025). Employment without adequate institutional support—such as sufficient maternity leave, affordable childcare, and flexible working arrangements—may contribute to role overload, understood here as strain arising when the demands of multiple social and occupational roles exceed available time and resources (Dukhaykh, 2023). Because these data are cross-sectional, they describe a snapshot rather than a trajectory and cannot establish whether reform has altered the burden of mental-health problems over time.

Few studies have integrated Saudi national surveillance data with a theoretically grounded and culturally specific analysis addressing the biological, psychological, social, and spiritual dimensions of women’s mental health simultaneously. Existing research has tended either to report epidemiological patterns without substantial theoretical integration or to propose conceptual frameworks without systematically anchoring their interpretation in national-level indicators. The present study addresses this gap through a multi-source secondary analytical design that integrates published aggregate indicators from four official Saudi national statistical sources with a structured, theory-informed synthesis of peer-reviewed evidence. It pursues four objectives: (1) to characterize gender disparities and patterns of anxiety and depression among Saudi women using the most current available national indicators; (2) to examine the biological, psychological, social, and spiritual determinants relevant to Saudi women across the life course, including reproductive transitions and the changing labor-market context; (3) to analyze structural and cultural barriers to mental-healthcare access, with particular attention to somatization, family-related stigma, and detection gaps; and (4) to develop a culturally integrated conceptual framework—the Islamic Biopsychosocial–Spiritual (BPSS-I) model—to inform future empirical research, culturally responsive clinical practice, and mental-health policy within the context of Saudi Vision 2030.

## 2 Materials and methods

### 2.1 Study design

This study employed a multi-source secondary analytical design to investigate women’s mental health in Saudi Arabia by integrating two complementary evidence streams: published aggregate indicators from four official Saudi national statistical sources and peer-reviewed empirical and theoretical evidence identified through a structured literature search. The design was developed to enable comparative analysis of national mental-health and reproductive-health indicators while situating the observed patterns within their broader biological, psychological, social, cultural, and spiritual contexts.

The primary national-data component involved the structured extraction, organization, and comparative analysis of published indicators from the four official Saudi sources. Gender-specific prevalence estimates, reproductive-health indicators, and measures relevant to mental-health service utilization were examined across the available national sources to identify patterns, disparities, and areas of convergence or divergence. Because the underlying individual-level microdata were not available, the analysis was conducted at the level of published aggregate indicators. No individual-level statistical modeling, pooled prevalence estimation, or re-analysis of the original survey microdata was undertaken. The analytical contribution therefore lies in the cross-source comparison and integrated interpretation of national indicators rather than in the generation of new estimates from individual-level data.

A structured, theory-informed evidence synthesis complemented the national-data analysis. Peer-reviewed empirical studies were used to contextualize the national findings, examine factors that may contribute to the observed patterns, and evaluate evidence relating to biological, psychological, social, cultural, and spiritual dimensions of women’s mental health. Foundational theoretical literature and relevant contextual sources were additionally used to support conceptual interpretation and the development of the proposed Islamic Biopsychosocial–Spiritual (BPSS-I) framework. The literature component therefore served as a complementary source of explanatory and theoretical evidence rather than constituting the primary study design.

The literature search incorporated predefined abstract-level eligibility criteria and a documented screening procedure to enhance transparency in evidence identification. It was not designed or conducted as a completed systematic review. As detailed in Section 2.4, full-text eligibility assessment was not completed for all potentially relevant records, and evidence used in the theory-informed synthesis was selected purposively according to its relevance to the predefined analytical domains. Supplementary theoretical, contextual, and official sources were also incorporated where necessary and were not treated as part of the screened evidence set. Accordingly, the structured literature search should not be interpreted as a systematic-review process or the retrievable records as a final systematic-review inclusion set.

The two evidence streams were integrated analytically to identify convergent and divergent patterns and to develop a multidimensional interpretation of women’s mental health in the Saudi context. This integration informed the development of the BPSS-I framework as a conceptual output of the study. The framework is proposed as a theory-informed analytical model for subsequent empirical testing and should not be interpreted as having been independently validated by the present analysis.

Because the study relied exclusively on publicly available aggregate statistics and previously published sources, involved no collection or analysis of identifiable individual-level data, and included no direct participation of human subjects, institutional ethical review was not required.

### 2.2 Official national data sources and their measures

Four official publications were used as primary sources; for each, we note the measurement basis, because the instruments differ in kind and are not directly comparable.

*Health Status Statistics 2025* (General Authority for Statistics, GASTAT) reports nationally representative, self-reported estimates of depression and anxiety severity (mild, moderate, severe) disaggregated by sex, age group, nationality, and administrative region, from the National Health Survey 2025 and the Women and Child Health Survey 2025. The bulletin reports self-rated severity categories and does not specify the exact screening instrument or cut-offs, which we treat as a limitation (Section 4.5).

*Women’s Health and Reproductive Care Statistics 2024 and 2025* (GASTAT) provide annual, nationally representative, self-reported data on reproductive-health indicators, maternal-care utilization, family planning, and health-decision-making autonomy among women aged 15–49 years, from the Women and Child Health Survey. These supply reproductive-context indicators rather than mental-health measures.

The Saudi National Mental Health Survey (SNMHS) Technical Report (King Salman Center for Disability Research, with King Faisal Specialist Hospital and Research Centre, the Ministry of Health, and King Saud University; international collaborators including the World Health Organization, Harvard Medical School, and the University of Michigan) provides the first nationally representative psychiatric-epidemiological estimates for the Kingdom. Fieldwork ran from 2010 to 2016 with a stratified random sample of 4,004 Saudi adults aged 15–65 years across all 13 administrative regions, using the Saudi-Arabic Composite International Diagnostic Interview 3.0 (CIDI 3.0), which yields diagnoses according to the Diagnostic and Statistical Manual of Mental Disorders, Fourth Edition (DSM-IV), administered by computer-assisted personal interview. Unlike the self-report GASTAT bulletins, the SNMHS uses a structured diagnostic interview; this distinction is central to our interpretation of the measured gender gap (Section 4.1).

### 2.3 Data extraction and synthesis

Aggregate estimates were extracted verbatim from the four publications and independently checked against the source documents; any discrepancy was resolved by returning to the original bulletin. No statistical re-analysis, pooling, or significance testing was performed on the national estimates, and no cross-source combined prevalence was computed, because the sources differ in instrument (self-report screening vs. structured diagnostic interview), survey year, and target population and are therefore not directly poolable. National estimates and literature findings were integrated narratively by domain -examining where independent sources converge (e.g., higher female risk across national and regional data) and where they diverge (e.g., self-report vs. diagnostic-interview gender ratios) -rather than by quantitative synthesis.

### 2.4 Literature search and selection

A structured literature search was conducted as a complementary component of the study to identify peer-reviewed evidence relevant to the interpretation of the national-data findings and the development of the study’s theoretical framework. The search was designed to provide a transparent and organized evidence base for contextual and explanatory synthesis; it was not intended to constitute a completed systematic review. Accordingly, Figure 1 summarizes literature identification, abstract-level screening, full-text retrieval, and subsequent narrative evidence synthesis. It should not be interpreted as a Preferred Reporting Items for Systematic Reviews and Meta-Analyses (PRISMA) flow diagram documenting a final systematic-review inclusion set.

**Figure 1:**
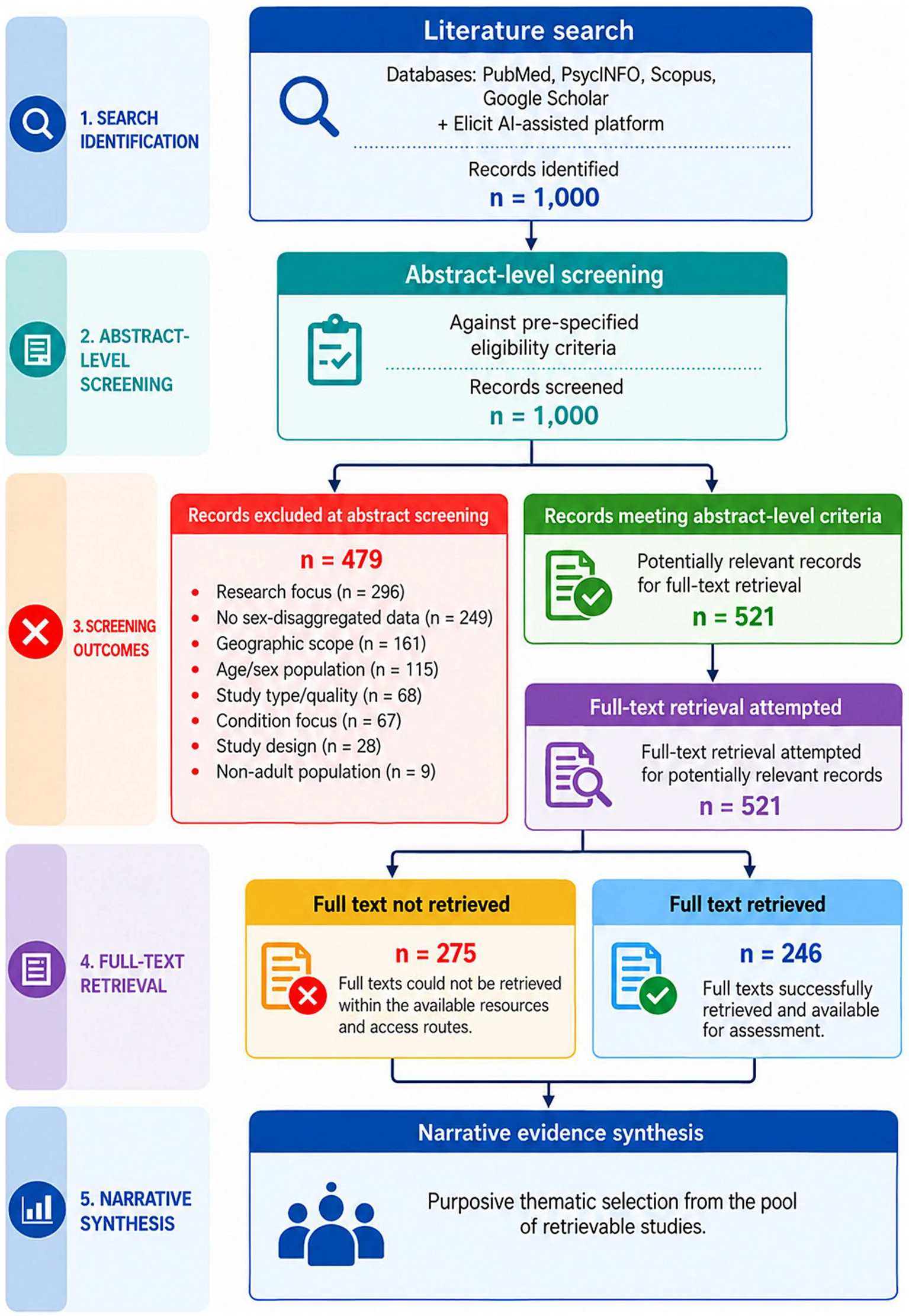
Literature identification, abstract-level screening, full-text retrieval, and narrative evidence synthesis. The figure summarizes the structured literature-search component of the study. Of 1,000 records screened at the abstract level, 521 met the abstract-level eligibility criteria; Full-text retrieval was attempted for these records, with 246 successfully retrieved and 275 not retrieved through the available resources and access routes. The 246 retrievable records represent an evidence pool rather than a final systematic-review inclusion set. Narrative evidence was selected purposively from this pool according to relevance to the predefined analytical domains; because this stage did not constitute a formal systematic-review full-text inclusion process, no systematic-review-style final inclusion count was generated. Supplementary theoretical, contextual, methodological, and official sources used elsewhere in the study were not necessarily part of the 1,000-record screening dataset. The four official Saudi national data sources constituted a separate evidence stream and were integrated with the narrative evidence during the analytical stage. Abstract-level exclusion criteria were not mutually exclusive.

The search covered PubMed, PsycINFO, Scopus, and Google Scholar and was supplemented by Elicit, an artificial intelligence (AI)-assisted literature platform. Search combinations incorporated population terms (e.g., “Saudi Arabia,” “GCC,” “Arab,” and “Muslim women”) and terms corresponding to the principal analytical domains, including depression, anxiety, gender differences, biopsychosocial determinants, postpartum depression, somatization, stigma, help-seeking, primary-care detection, Islamic religiosity, religious coping, culturally adapted interventions, and the mental-health implications of Saudi Vision 2030. Search terms were adapted to the syntax and functionality of each database. Detailed search strategies are provided in the Supplementary Material. As described in Section 2.6, Elicit assisted with search-query formulation, literature retrieval, organization, and abstract-level screening. All screening decisions and classifications used in the study were reviewed and verified by the authors.

Records were considered potentially eligible if they comprised peer-reviewed empirical studies, systematic reviews, meta-analyses, or theoretical papers published in English or Arabic between 1990 and 2026, with priority during evidence synthesis given to research published after 2010. Potentially eligible records addressed mental-health outcomes, determinants, help-seeking, or culturally relevant mechanisms among women in Saudi Arabia, other Gulf Cooperation Council (GCC) countries, or Muslim-majority Arab populations considered contextually informative for the Saudi setting. Where the analytical question concerned gender differences, sex-disaggregated findings were required. Records were excluded at abstract-level screening when they lacked relevance to the study questions or population, fell outside the geographical or thematic scope, did not provide sex-disaggregated information where required, or failed to meet the eligible publication or study-type criteria.

The search identified 1,000 records, all of which underwent abstract-level screening against the predefined criteria. A total of 479 records were excluded at this stage, leaving 521 records that met the abstract-level criteria and were considered potentially relevant for full-text retrieval. The criterion-specific exclusion counts presented in Figure 1 are not mutually exclusive; individual records could meet more than one exclusion criterion. These counts therefore indicate the frequency with which individual exclusion criteria were identified and should not be summed to calculate the total number of excluded records.

Full-text retrieval was attempted for all 521 potentially relevant records. Full texts for 275 records could not be retrieved through the resources and access routes available to the authors, leaving 246 records for which full texts were successfully retrieved and available for assessment. The 246 records therefore represent the retrievable evidence pool rather than a final set of studies formally included through a completed systematic-review process. Because full-text retrieval was incomplete and the subsequent synthesis was purposive and thematic rather than based on a formal full-text inclusion stage, no definitive systematic-review-style final inclusion count was generated.

The narrative evidence synthesis drew purposively from the pool of retrievable studies according to their relevance to the predefined analytical domains and their capacity to contextualize or explain patterns identified in the national-data analysis. Priority was given to Saudi-specific evidence, followed by evidence from other GCC settings and, where Saudi or GCC evidence was limited, contextually relevant research from Muslim-majority Arab populations. Within each domain, greater interpretive weight was given to nationally representative studies, systematic reviews and meta-analyses, larger empirical studies, and evidence directly addressing women or reporting sex-disaggregated findings. Evidence from outside Saudi Arabia was used principally to contextualize mechanisms for which Saudi-specific evidence remained limited and is identified as such in the manuscript. Selection at this stage was therefore based on analytical relevance and evidentiary contribution rather than on a formal systematic-review inclusion procedure.

In addition to the screened literature pool, supplementary sources were used where necessary to support the conceptual, theoretical, cultural, methodological, and policy dimensions of the analysis. These included foundational theoretical works, relevant reviews, selected contextual studies, and official national and international sources identified through supplementary searching and citation tracing. Such supplementary sources were not necessarily represented among the 1,000 records subjected to abstract-level screening and should not be interpreted as having passed through that screening process. The four official Saudi national statistical sources constituted the separate national-data component described in Sections 2.1–2.3 and were integrated analytically with the published evidence rather than treated as records within the literature-screening dataset.

Evidence synthesis was narrative and theory-informed rather than meta-analytic. Because the literature component involved purposive thematic selection and incomplete full-text retrieval rather than a completed systematic-review process, it was intended to support interpretation and theory development rather than to produce an exhaustive or strictly reproducible systematic estimate of the literature. No formal risk-of-bias assessment, meta-analysis, or quantitative evidence synthesis was undertaken. Instead, evidence was interpreted with attention to consistency across sources, study design, methodological limitations, source type, and contextual relevance to Saudi Arabia. The implications of these methodological characteristics for interpretation and reproducibility are addressed explicitly in Section 4.5.

The abstract-level screening decisions and criterion-specific classifications for all 1,000 screened records are provided in the Supplementary Material.

### 2.5 Organizing framework

To structure the synthesis, we use an author-proposed organizing framework, the Islamic Biopsychosocial–Spiritual (BPSS-I) model, developed for this article as a conceptual extension of Engel’s (1977) biopsychosocial model. The framework is used as a heuristic for organizing heterogeneous evidence and generating testable hypotheses rather than as an empirically validated or prespecified causal model; its formal testing is identified as a priority for future research (Section 4.4). Although biopsychosocial-spiritual (BPSS) models have previously incorporated spirituality as an additional dimension of mental-health assessment, the BPSS-I framework adapts this broader tradition to the contemporary Saudi context by specifying culturally and gender-relevant mechanisms within four interacting domains.

The biological domain encompasses reproductive and life-course conditions that may shape women’s mental health, including pregnancy, the postpartum period, infertility, and menopause. The psychological domain encompasses anxiety and depression as well as the ways psychological distress may be experienced, interpreted, expressed, and managed, including somatization and coping processes. The social domain foregrounds family-system dynamics and gender-specific structural determinants, including spousal and extended-family support, family involvement in healthcare decisions, honor-based stigma, mobility, employment, and changing social and occupational roles. Honor-based stigma is defined here as stigma arising when mental-health disclosure is perceived as potentially threatening the reputation or social standing of the individual and her family. The social domain also incorporates transformations associated with Saudi Vision 2030, particularly changes in women’s labor-force participation, mobility, and social roles. The spiritual domain incorporates Islamic concepts as potential explanatory constructs, including *nafs* (the self, encompassing different states of inner moral and psychological experience), *sabr* (patient perseverance in responding to adversity), *tawakkul* (reliance on God), and *dhikr* (remembrance of God through devotional practice), together with positive and negative forms of religious coping. This formulation treats spirituality as a culturally situated domain rather than as an undifferentiated generic construct (Sa’ari and Chik, 2025; Keshavarzi and Haque, 2013).

The distinctive contribution of BPSS-I lies not simply in adding these determinants to the conventional biopsychosocial model but in examining their potential interaction. Family systems, for example, may operate simultaneously as sources of emotional and practical support and as influences on disclosure and professional help-seeking (Fakhr El-Islam, 2008; Scull et al., 2014). Similarly, changing opportunities for employment and mobility may increase social participation and access to resources while also generating role strain when occupational and domestic demands exceed available family or institutional support. Religious coping may likewise function differently depending on whether distress is interpreted through supportive practices and meanings or through negative interpretations such as spiritual deficiency or divine punishment. The framework therefore does not assign any social, cultural, or spiritual determinant an inherently protective or harmful role; its proposed effect depends on its interaction with other domains and with the woman’s particular life-course and social context.

A practical example illustrates this interaction. Postpartum psychological distress may involve a biological reproductive transition; psychological symptoms such as anxiety, depressed mood, or somatic expressions of distress; social conditions such as spousal and extended-family support, family involvement in healthcare decisions, and the perceived acceptability of seeking professional care; and spiritual processes through which distress is interpreted and managed. BPSS-I proposes that these dimensions should be considered together rather than treated as independent explanations. In the same way, the mental-health implications of women’s changing social and occupational roles under Saudi Vision 2030 may depend on the interaction between expanded opportunities, family-role renegotiation, institutional support, coping processes, and access to appropriate mental-healthcare services.

Accordingly, BPSS-I is applied in this study as an interpretive scaffold for organizing and examining the assembled evidence and for identifying potential cross-domain pathways that can be tested in subsequent research. It is not intended to establish causal relationships from the present secondary analysis. Its adequacy—including areas where the available evidence is limited, derives from non-Saudi populations, or does not clearly support the proposed relationships—is revisited in the Discussion. Figure 2 provides a schematic representation of the four interacting domains, their principal Saudi-context determinants, and the hypothesized pathways through which they may jointly shape women’s mental-health outcomes and help-seeking.

**Figure 2:**
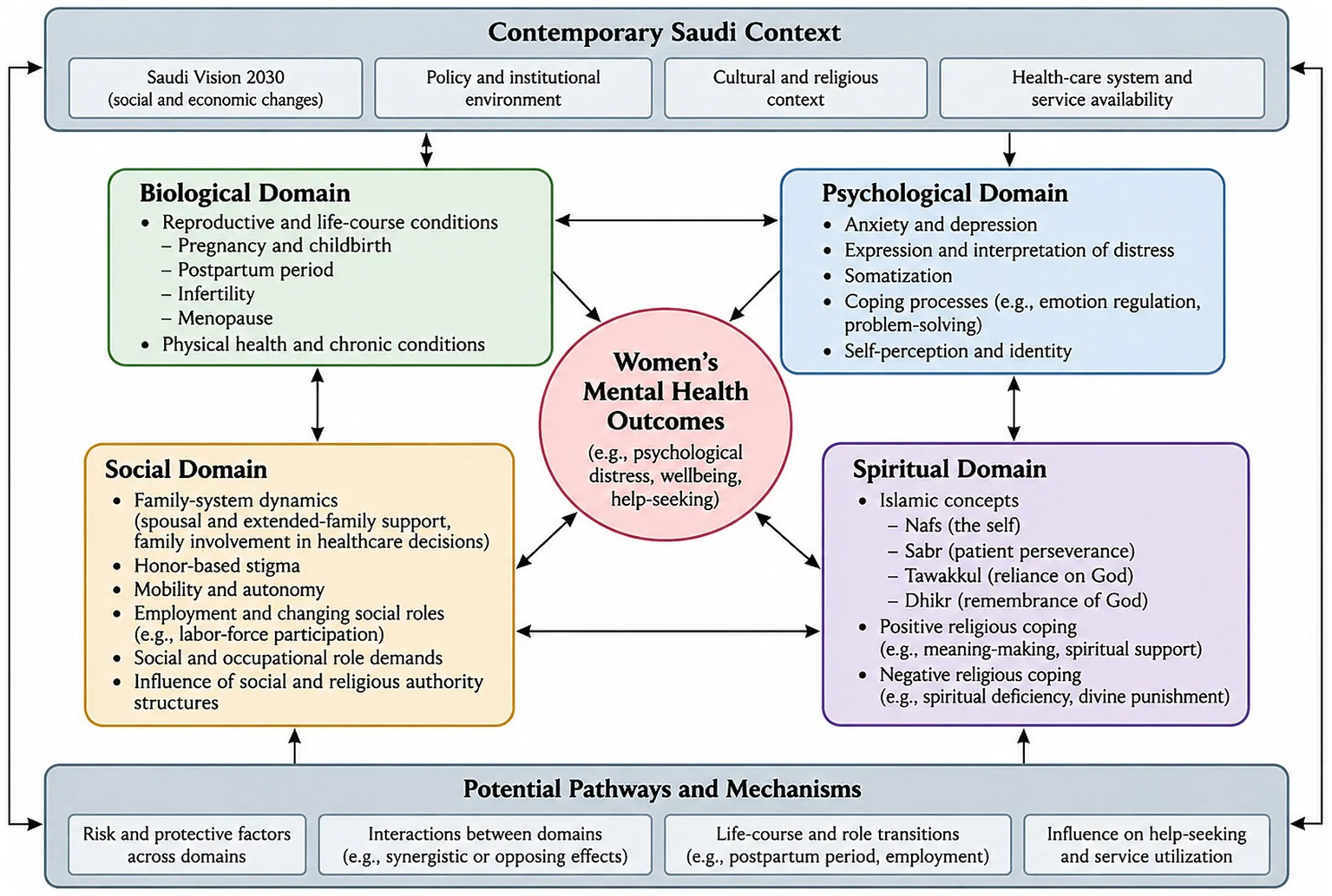
The Islamic biopsychosocial–spiritual (BPSS-I) framework for women’s mental health in the saudi context.

### 2.6 Use of generative artificial intelligence

During the preparation of this manuscript, the authors used Elicit to assist with literature retrieval, abstract-level screening according to predefined eligibility criteria, reference verification, and preparation of the search and screening summary. Claude (Anthropic, Sonnet family) was used for academic language refinement and editorial improvement. All AI-generated outputs, including screening classifications, reference verification, and edited text, were reviewed, verified, and, where necessary, revised by the authors before incorporation into the manuscript. The authors take full responsibility for the accuracy, integrity, and intellectual content of this publication.

## 3 Results

### 3.1 Prevalence and gender distribution of anxiety and depression

#### 3.1.1 National surveillance data

The Health Status Statistics 2025 show a consistent female excess in self-reported anxiety and depression among Saudi adults aged 15 years and above. Severe anxiety was reported by 9.0% of women versus 6.9% of men (a female-to-male ratio of about 1.3:1), and moderate anxiety by 1.1% of women versus 0.8% of men. For depression, moderate-severity depression was reported by 0.2% of women versus 0.1% of men, while mild depression was comparable across sexes (0.7% in both). At the same time, 99.2% of adults self-rated their general health as good or better -a pattern consistent with the well-documented tendency toward positive health self-assessment in high-stigma settings, and one reason these self-report figures should be read as a lower bound rather than a true prevalence (General Authority for Statistics, 2025a).

On social connectedness -a recognized protective factor −71% of adults aged 50 and above reported sufficient contact with people they care about, with near-identical rates for women (71.1%) and men (70.9%). Across the adult population, 39.3% reported a routine health checkup, higher among Saudi nationals (44.2%) than non-Saudi residents (35.0%) (General Authority for Statistics, 2025a).

These self-report figures sit within the broader epidemiological picture from the Saudi National Mental Health Survey (SNMHS), which estimated that 34% of Saudi citizens met criteria for a mental disorder at some point in life, rising to roughly two in five (about 40%) among those aged 15–24 (Altwaijri et al., 2019). Because the GASTAT bulletins rely on self-rated severity categories while the SNMHS uses a structured diagnostic interview, the two are not directly comparable and are best read as complementary lower- and diagnostic-level estimates (Section 4.1). Table 1 summarizes the national indicators.

**Table 1:** Mental health indicators among Saudi adults from official national data sources.

Indicator	Women	Men	Total	Source (instrument)
Severe anxiety	9.0%	6.9%	—	General Authority for Statistics (2025a) (self-report)
Moderate anxiety	1.1%	0.8%	—	General Authority for Statistics (2025a) (self-report)
Moderate depression	0.2%	0.1%	—	General Authority for Statistics (2025a) (self-report)
Lifetime mental disorder (any)	—	—	34%	Altwaijri et al. (2019) (CIDI 3.0)
Lifetime disorder, ages 15–24	—	—	~40%	Altwaijri et al. (2019) (CIDI 3.0)
Ever sought treatment	—	—	13.6%	Altwaijri et al. (2019) (CIDI 3.0)
Severe cases receiving no treatment	—	—	80%	Altwaijri et al. (2019) (CIDI 3.0)

GASTAT figures are self-reported severity categories; SNMHS figures are structured-interview (CIDI 3.0) estimates reported for the total sample, not sex-disaggregated. The two sources use different instruments and are not directly comparable.

#### 3.1.2 Comparative regional evidence

The Saudi pattern aligns with the wider Gulf, though cross-study comparison is limited by differing instruments and sampling. A meta-analysis of 18,717 GCC adults reported broadly stable depression prevalence over two decades and consistently identified younger and female individuals as higher-risk groups (Camia and Alhallami, 2023). Globally, the female excess is robust: WHO collaborative research across 15 centers on four continents found women had roughly 1.6 times the odds of current depression and panic disorder relative to men (Gater et al., 1998), and a 122-country analysis reported that the size of the female-to-male depression gap tracks structural gender inequality independently of national income (Yu, 2018). The Gulf “paradox” -improving formal gender indicators alongside persistent informal constraints on autonomy, mobility, and help-seeking -may explain why standard inequality indices understate the structural burden on Saudi women’s mental health (Table 2).

**Table 2:** Biopsychosocial-spiritual risk and protective factors for mental health among Saudi women (only factors supported by the cited sources are listed).

Domain	Risk factors	Protective factors	Key sources
Biological	Postpartum period; infertility; menopause	Skilled antenatal care	General Authority for Statistics (2024, 2025b); Zarroug et al. (2025); Kandasamy et al. (2024)
Psychological	Somatization; supernatural attribution; emotional divorce	Emotional disclosure; positive coping	El-Rufaie et al. (1999); Al-Shahrani and Hammad (2023)
Social	Family conflict; poor spousal support; restricted mobility; familialism-based stigma; role overload	Social support; social interaction; family involvement	Scull et al. (2014); Alghamdi et al. (2026); Alshahrani et al. (2023)
Spiritual	Negative religious coping; spiritual struggle; illness attributed to divine punishment	Positive religiosity; *tawakkul*; *dhikr*; *dua*; religious community	Fekih-Romdhane et al. (2023); Francis et al. (2019); Adil et al. (2022); Simonovich et al. (2022)

### 3.2 Biopsychosocial-spiritual determinants of women’s mental health

#### 3.2.1 Biological domain: reproductive and maternal health

The Women’s Health and Reproductive Care Statistics for 2024 and 2025 describe the reproductive context in which these vulnerabilities are embedded. In 2024, 71.4% of deliveries were vaginal and 28.1% cesarean; 99.5% of mothers attended at least one antenatal visit (mean 7.57 visits), skilled personnel attended 99.6% of deliveries, and 66.1% of births occurred in government hospitals. By 2025, 89.6% of mothers attended at least four antenatal visits (mean 7.8), and 68.2% of deliveries occurred in government facilities (General Authority for Statistics, 2024, 2025b).

Against this backdrop, the peer-reviewed literature documents a substantial postpartum depression burden. Two recent systematic reviews synthesized 22 and 32 Saudi studies respectively; reported prevalence varied widely with setting and diagnostic threshold, from about 15% in some Jeddah samples to 76% in a rural Jazan sample (Mukrim et al., 2025; Zarroug et al., 2025; Abdelmola et al., 2023). Urban estimates were intermediate (Riyadh studies ~25%–38%). Across these reviews, the most consistent correlates were psychosocial rather than biological: limited family support, poor spousal support, and unplanned pregnancy were the dominant associated factors, outweighing delivery mode or hormonal status (Mukrim et al., 2025). Family conflict was associated with roughly six times higher odds of postpartum depression, and an unsupportive spouse with a two- to fourfold higher odds, depending on region (Alshahrani et al., 2023; Al Nasr et al., 2020).

For menopausal women, a cross-sectional survey in the Asir region found 65% reported depressive symptoms and 52% anxiety symptoms, with widowed or separated status and illiteracy the strongest independent correlates (Kandasamy et al., 2024). Infertility carries additional psychological burden: infertile Kuwaiti women scored significantly higher than pregnant controls across all Hospital Anxiety and Depression Scale domains, including depression, self-blame, and suicidal ideation (Fido and Zahid, 2004).

#### 3.2.2 Psychological domain: somatization and supernatural attribution

A defining feature of how distress presents among Arab women clinically is somatization -expression through physical rather than affective complaints. In Arab primary-care settings, somatized mental disorder has been estimated to account for about 48% of psychiatric presentations (El-Rufaie et al., 1999), with headache, backache, and abdominal pain the dominant cluster (Eid et al., 2025). Shafi and Shafi (2014) describe this as “ticket behavior”: a socially acceptable somatic complaint that grants entry to care while the psychological driver stays undisclosed. Arabic carries a rich affective vocabulary for distress, including *kaaba* and *muraqiya* from medieval Islamic medicine (Youssef, 1994), yet norms against emotional disclosure -especially among older women, for whom composure is an identity-constitutive value -suppress its use in clinical encounters.

Supernatural attribution represents an important pathway through which psychological distress may be interpreted outside the biomedical model, potentially delaying or diverting women from professional mental-health care. In the UAE, focus-group research found that many postpartum women did not recognize postnatal depression as a psychological condition, instead attributing their symptoms to the evil eye or jinn (Ghubash and Eapen, 2009). Similarly, among infertile women in Kuwait, participants with lower educational attainment were more likely to attribute infertility-related distress to witchcraft or divine retribution and to seek help from faith healers before consulting mental-health professionals (Fido and Zahid, 2004). Together, these findings illustrate how supernatural explanatory models may influence help-seeking behaviour in parts of the Gulf region, although comparable contemporary evidence from Saudi Arabia remains limited.

#### 3.2.3 Social domain: family systems, collectivism, and structural barriers

The family system is the primary mediator of social determinants here, acting simultaneously as chief source of support and chief gatekeeper of help-seeking. Extended kinship networks absorb mental illness -moderating the worst outcomes -but mean professional care is sought only after family management fails, typically at a more severe, chronic stage (Fakhr El-Islam, 2008). For women specifically, seeking psychiatric consultation without family involvement can draw family sanction, since healthcare decisions are often made on a woman’s behalf in a relational rather than individualist model of identity (Carter and Rashidi, 2003).

The “emotional divorce” phenomenon -cohabiting estrangement without legal dissolution -illustrates the structural pressures women face. In a cross-sectional study of 241 married Saudi women, emotional divorce accounted for 61% of the variance in combined depression, anxiety, and loneliness scores; because legal divorce is socially and economically costly for women, many remain in psychologically dissolved marriages (Al-Shahrani and Hammad, 2023). Post-2018 reform data partially soften this picture: in a study of the post-reform period, mobility, social interaction, and perceived social support were each independently associated with lower depression risk, whereas possession of a driving licence was not independently associated once broader mobility and social connectedness were accounted for -suggesting the benefit of reform operates through social access rather than the formal right itself (Alghamdi et al., 2026).

#### 3.2.4 Spiritual domain: religiosity as protective and risk factor

Saudi-specific empirical evidence on the relationship between Islamic religiosity, religious coping, and women’s mental health remains limited. Accordingly, this domain draws cautiously on evidence from other Gulf, Muslim-majority, and broader Arab Muslim populations to identify potentially relevant mechanisms rather than to assume that findings from these populations are directly transferable to Saudi women.

Across this broader evidence base, Islamic religiosity appears to operate as a context-dependent determinant whose association with mental health varies according to the form of religious coping involved. Among Emirati female college students, religiosity was negatively correlated with depressive symptoms, and women with severe depression reported lower religiosity than asymptomatic peers (Thomas et al., 2016). Evidence from a Pakistani Muslim sample further suggests that *tawakkul* (reliance on God) may mediate the relationship between intrinsic religious orientation and lower depressive affect (Adil et al., 2022). In the perinatal context, a synthesis of nine studies involving Muslim populations found that practices including *salah* (ritual prayer), *dua* (supplication), and Qur’anic recitation were associated with lower perinatal anxiety, depression, and pain (Simonovich et al., 2022).

The distinction between positive and negative religious coping is particularly important. In a multi-religious study, negative religious coping—including attributing illness to divine punishment, experiencing spiritual struggle, or questioning God’s love—independently predicted both anxiety and depression, whereas positive coping showed no independent effect after full adjustment (Francis et al., 2019). This distinction may also have clinical relevance: when depression is interpreted as evidence of spiritual deficiency, an appropriate response may need to address such religious interpretations rather than assume that religiosity is uniformly protective.

Evidence from broader Arab populations further suggests that stigma may modify the relationship between religiosity and help-seeking. Among 9,782 Arab Muslim participants across 16 countries, higher religiosity was associated with more favorable help-seeking attitudes when stigma was low to moderate, but this association disappeared at high levels of stigma (Fekih-Romdhane et al., 2023). Taken together, these non-Saudi findings identify potentially relevant mechanisms but should not be interpreted as direct evidence of equivalent relationships among Saudi women. Instead, they provide comparative evidence for hypotheses that require Saudi-specific empirical testing. Within the present framework, religiosity is therefore treated neither as inherently protective nor as inherently harmful, but as a potentially modifying influence whose mental-health implications depend on the form of religious coping, the interpretation of distress, and the surrounding social and stigma context.

### 3.3 The treatment gap and the detection gap

#### 3.3.1 The treatment gap

The distance between disorder prevalence and treatment receipt in Saudi Arabia is among the largest documented. The SNMHS found that although about 34% of citizens receive a lifetime diagnosis, only 13.6% ever seek treatment, and 80% of those with severe disorders receive no care of any kind (Altwaijri et al., 2019). This is a treatment-access gap -distinct from, though compounded by, the detection problem described below.

#### 3.3.2 Stigma and familialism

Stigma is a consistently identified barrier to mental-health help-seeking across Saudi Arabia and other GCC settings, including the UAE, Kuwait, and Qatar. In the present study, *familialism* refers to the influence of collective family interests, reputation, and social standing on individual health decisions, including decisions about disclosing psychological distress and seeking professional care. This relational orientation can provide important sources of support, but it may also amplify stigma when mental-health disclosure is perceived as carrying consequences for the wider family. In Kuwait, grounded-theory interviews identified stigma, gossip, and reputation as interrelated drivers of help-avoidance, with concerns about family honor and marriageability appearing more salient than cost or service availability (Scull et al., 2014). Saudi-specific evidence likewise documents substantial stigma: a nationally representative study across all 13 regions (*n* = 4,533) found that male gender, lower education, and no personal contact with a person with mental illness predicted higher stigma. Respondents were also more optimistic about recovery than about social acceptance, suggesting a potential role for recovery-focused anti-stigma interventions (BinDhim et al., 2024).

Broader Arab evidence provides additional comparative context but should not be assumed to represent Saudi women directly. The multinational IMPACT Project (*n* = 10,036 across 16 Arab countries) found that 26.5% of participants held stigmatizing attitudes, 31.7% had poor mental-health knowledge, and 28.0% reported negative attitudes toward professional help-seeking (Fekih-Romdhane et al., 2022). Across the wider literature, women have generally shown greater willingness than men to seek professional help, while structural constraints—including family involvement in healthcare decisions, financial dependency, and mobility restrictions—may still limit access to care. This combination of greater stated willingness to seek help and practical constraints on accessing services illustrates an important dimension of the gender-specific treatment gap (Gearing et al., 2014; Ciftci et al., 2013).

#### 3.3.3 The detection gap: somatization in primary care

The diagnostic side of the gap runs largely through somatization. In Riyadh primary care, physicians’ agreement with standardized diagnoses was only fair for depression and slight for somatized presentations -both below acceptable clinical thresholds (Becker et al., 2004). A Kuwait primary-care study (*n* = 1,046) found 42.3% of attenders had at least one mental condition, 34.4% had mental-physical comorbidity, and somatization was the most frequent comorbid disorder regardless of physical illness (Alkhadhari et al., 2018). Among women in Riyadh gastroenterology clinics, 44% screened positive for depression and 34% for anxiety, yet most subsequently sought help at private clinics or from religious practitioners rather than mental health services -reflecting stigma-driven avoidance of public psychiatric labeling (Alosaimi et al., 2014).

### 3.4 Vision 2030 reforms and women’s mental health

#### 3.4.1 Labor market transformation

Saudi women’s labor-force participation rose from roughly 19% in 2017 to over 35% by 2022, with the steepest gains in urban regions, exceeding the Vision 2030 target of 30% ahead of schedule; private-sector female employment had already risen sharply before the headline reforms (Dukhaykh, 2018; Alghamdi et al., 2026).

#### 3.4.2 Mental health outcomes of reform

The mental-health evidence does not support a simple “empowerment improves wellbeing” narrative, though the available studies are cross-sectional and cannot establish direction. Saudi women in 2023 reported general life satisfaction alongside elevated psychological distress, with depression highest among younger and more highly educated women (Almadani and Alwesmi, 2023). Among 580 working women in Riyadh, about half screened positive for mild-to-moderate depression and anxiety, and 65% reported needing longer maternity leave than they received (Alateeq et al., 2025). A structural-equation study of 593 Saudi women in the post-reform period found mobility, social interaction, and social support associated with lower depression risk, while driving-licence possession was not independently associated (Alghamdi et al., 2026). An analysis of national datasets described a “diagnostic skew” in which psychotic disorders are overrepresented in service data while emotional distress -the predominant presentation among women -is under-documented, so any population-level improvement in women’s wellbeing could be masked by existing health-information systems (Almakrob and Alduais, 2025). On present evidence, the labor reforms appear to have expanded formal rights without a proportionate reduction in psychological burden -a pattern consistent with role overload absent adequate institutional support, though this interpretation awaits longitudinal testing.

## 4 Discussion

This study integrates published aggregate indicators from four official Saudi national statistical sources with a structured, theory-informed synthesis of peer-reviewed evidence to examine women’s mental health across biological, psychological, social, and spiritual domains. The integrated analysis identifies a set of interrelated vulnerabilities that may contribute to what can be described as *structured invisibility*: Saudi women appear to experience a disproportionate burden of anxiety and depression, while sociocultural and structural factors may simultaneously constrain the recognition and disclosure of psychological distress and access to appropriate mental-healthcare services. This interpretation should be considered in light of the methodological characteristics and limitations of the study, particularly the heterogeneity of the underlying evidence, reliance on published aggregate national indicators rather than individual-level microdata, and predominance of cross-sectional evidence, all of which limit causal inference. Four themes warrant extended discussion: the gap between measured and likely underlying burden; the culturally specific mechanisms shaping the treatment gap; the mental-health implications of Saudi Vision 2030 reforms; and the design principles—and evidentiary limits—of culturally integrated interventions.

### 4.1 Measured versus likely actual burden

The national data present an apparent tension: 99.2% of Saudi adults rate their health as good or better, yet the SNMHS estimated that 34% of citizens meet lifetime criteria for a mental disorder, only 13.6% ever seek treatment, and 80% of those with severe disorders receive no care (General Authority for Statistics, 2025a; Altwaijri et al., 2019). This is less a contradiction in the data than a signature of measurement in high-stigma contexts. Self-rated health in collectivist societies with strong norms of composure and endurance tends to index social identity management as much as clinical status (Halbreich et al., 2007).

The gender-specific dimension is that the female-to-male ratio in *self-reported* anxiety and depression in the Saudi data (about 1.3:1) is compressed relative to the ratios typically seen with structured diagnostic interviews -for instance, the WHO 15-centre collaborative study, where women had roughly 1.6 times the odds of current depression and panic disorder (Gater et al., 1998). Rather than indicating genuinely lower female vulnerability in Saudi Arabia, this compression is consistent with the measurement paradox described by Alansari (2006) across 16 Islamic countries: where norms constrain emotional expression across both sexes, and where women disproportionately present somatically rather than affectively, self-report instruments undercount female burden relative to clinician-administered interviews.

The somatization evidence supports this reading. Somatized mental disorder accounts for roughly 48% of psychiatric presentations in Arab primary care (El-Rufaie et al., 1999), and Saudi primary-care physicians show only slight agreement with standardized criteria for somatized depression (Becker et al., 2004). When women route distress through gastroenterology, orthopedics, and general medicine rather than mental health services, they become invisible to psychiatric epidemiology even as they accumulate investigations that never address the underlying condition. The “ticket behavior” of Shafi and Shafi (2014) is not irrational from the patient’s standpoint -it secures care while avoiding the stigma of a psychiatric label -but it systematically depresses prevalence estimates in surveys built on self-reported affective symptoms.

The implication is that the true gender gap in Saudi women’s mental-health burden is plausibly larger than surveillance data suggest, so a policy response anchored only to official prevalence figures would likely underinvest relative to actual need. This is not a criticism of the national statistical infrastructure -among the most rigorous in the Arab world -but a limitation of self-report measurement that calls for supplementation with clinician-administered instruments and proxy indicators such as service utilization, somatic-disorder rates, and postpartum depression data.

### 4.2 The cultural specificity of the treatment gap

The 80% treatment gap among Saudis with severe disorders (Altwaijri et al., 2019) is not explained by service scarcity alone; the Kingdom maintains a substantial and growing mental-health infrastructure, including government psychiatric hospitals, general mental-health clinics, community units, and expanding digital services (Altwaijri et al., 2019). The gap is primarily cultural and structural, and its mechanisms are gender-differentiated in ways the literature has only recently specified.

The familialism mechanism described by Scull et al. (2014) in Kuwait offers the most parsimonious account of how collectivist organization converts generic stigma into specific help-avoidance. In individualist settings, stigma operates mainly through personal shame; in collectivist Gulf settings, the salient unit of identity is the family, so disclosure threatens the family’s reputation, marriage prospects, and standing -making concealment structurally rational. A woman consulting a psychiatrist without family knowledge risks not only personal stigma but sanction extending to her family’s social capital. This predicts that anti-stigma campaigns modeled on Western individual-education approaches will have limited traction in Gulf contexts unless they also address family-level reputation and the community honor economy within which disclosure is embedded.

The religion–stigma–help-seeking relationship reported by Fekih-Romdhane et al. (2023) adds a further layer. Because higher religiosity predicted more favorable help-seeking attitudes at low-to-moderate stigma but not at high stigma, religious framings of care -Islamic obligations to seek treatment, the Prophetic teaching that God has sent down a cure for every disease, the Quranic injunction against self-harm -can function as facilitators, but only where reputational risk does not overwhelm other considerations. Sequencing therefore matters: destigmatization should precede or accompany religiously framed health promotion rather than assuming Islamic legitimation alone can overcome familialism-based avoidance.

The gender asymmetry complicates the picture further. Women consistently report more positive help-seeking attitudes than men across GCC datasets (Fekih-Romdhane et al., 2022), yet face greater practical barriers to care. This “greater willingness, greater barriers” paradox reflects the distinction between attitudinal stigma and structural barriers articulated by Gearing et al. (2014). For women, the barriers lie less in their own beliefs than in family gatekeeping, financial dependency, restricted mobility, and fear of discovery. Addressing the female treatment gap therefore requires structural, not merely attitudinal, interventions: family-inclusive service models, digital and anonymous consultation that bypasses gatekeeping, and financial mechanisms that reduce economic dependency as a precondition for access.

### 4.3 Vision 2030 and the role-overload paradox

The Vision 2030 labor reforms mark the most significant change in Saudi women’s social position in the Kingdom’s modern history, and their mental-health implications are more nuanced than either optimistic or pessimistic readings allow. A gender-roles perspective suggests that reductions in traditional gender-role expectations across cohorts may contribute to narrowing the female depression gap over time (Seedat et al., 2009). From this perspective, structural empowerment would be expected eventually to reduce the female burden of anxiety and depression. The Saudi evidence from 2023 to 2025 suggests that any such benefit has not yet emerged and may be offset in the short term by role overload; however, all of these studies are cross-sectional and cannot establish temporal or causal relationships.

The role-overload paradox is well described in transitional-economy research: labor-market entry improves economic autonomy and self-esteem but, absent redistribution of domestic work and institutional support for work–family balance, raises total role demands and can accompany greater psychological distress (Dukhaykh, 2023; Almadani and Alwesmi, 2023). In Saudi Arabia the paradox is amplified by context: domestic expectations have not been renegotiated to reflect women’s changed labor roles, about 65% of working women report needing longer maternity leave than they receive, and affordable childcare remains structurally insufficient (Alateeq et al., 2025; Saad and Al-Kharif, 2026). The “diagnostic skew” described by Almakrob and Alduais (2025) -service data overrepresenting psychotic disorders and undercounting depression and anxiety -means that any genuine improvement in working women’s wellbeing could be masked within existing health-information systems, making population-level evaluation of reform effects difficult with current data.

That mobility and social connectedness, but not driving-licence possession, were associated with lower depression in the post-reform period (Alghamdi et al., 2026) converges with Wheeler’s (2020) analysis that the guardianship system, rather than the driving ban, was the primary structural barrier to women’s wellbeing. This suggests that the mental-health dividend of reform will depend less on formal legal change than on whether the surrounding social infrastructure—family role renegotiation, institutional childcare, workplace flexibility, and community acceptance of women’s economic independence—changes at a commensurate pace. A cross-sectional analysis of the 2022 Bangladesh Demographic and Health Survey (Alam et al., 2026), in which rejecting violence norms and greater social independence were associated with lower anxiety and depression, whereas greater decision-making autonomy was associated with higher anxiety, is consistent with the possibility that rapid role expansion without adequate social and structural support may generate new psychological burdens before delivering the anticipated mental-health benefits.

### 4.4 The Islamic biopsychosocial–spiritual framework: contributions, scope, and limitations

The BPSS-I framework proposed in this study is intended primarily as a Saudi-context organizing heuristic for understanding women’s mental health rather than as an empirically validated or universally applicable model. Its contribution lies in extending the biopsychosocial approach in three related directions. First, it reconceptualizes the social domain beyond generic demographic characteristics by incorporating family-system dynamics, including the potentially dual role of extended families as sources of support and influences on disclosure and help-seeking; honor-based stigma; changing gender and occupational roles; and the broader social transformations occurring under Saudi Vision 2030. Second, it operationalizes the spiritual domain through Islamic concepts and forms of religious coping, including the *nafs* framework distinguishing *nafs al-ammarah*, *nafs al-lawwamah*, and *nafs al-mutma’innah* as conceptual analogues of different inner moral and psychological states (Sa’ari and Chik, 2025; Keshavarzi and Haque, 2013), rather than assuming that generic spirituality constructs are interchangeable with Islamic meanings and practices in clinical settings (Al Ghaferi et al., 2017). Third, it applies an explicit gender lens to identify structural and relational determinants of women’s mental health that remain less explicit in the original gender-neutral formulation of the biopsychosocial model (Andermann, 2010; Christodoulou, 2010).

The Saudi focus of BPSS-I is deliberate. Although some of its constituent mechanisms—such as family involvement, religious coping, stigma, and collectivist forms of social organization—may also be relevant in other Gulf, Arab, or Muslim populations, the framework should not be assumed to transfer unchanged across these settings. Arab and Muslim populations are heterogeneous with respect to family organization, religious practice, socioeconomic conditions, gender norms, legal environments, healthcare systems, migration patterns, and the availability and acceptability of mental-health services. Evidence drawn from non-Saudi populations in the present synthesis is therefore used comparatively to illuminate potentially relevant mechanisms where Saudi-specific evidence is limited, not to imply cultural equivalence or to establish that the same mechanisms operate with the same direction or magnitude in Saudi women.

Accordingly, application of BPSS-I beyond Saudi Arabia would require context-specific adaptation and empirical validation rather than simple extrapolation. The four-domain architecture—biological, psychological, social, and spiritual—may provide a transferable analytical scaffold, but the content and relative importance of determinants within each domain should be established locally. For example, family involvement may function primarily as a protective resource in one setting but may constrain disclosure or autonomous help-seeking in another; religious coping may similarly be protective, neutral, or distress-amplifying depending on its form, interpretation, and surrounding social context. BPSS-I should therefore be understood as a Saudi-grounded framework with potentially adaptable components, not as a single model presumed to characterize Arab or Muslim women as culturally homogeneous populations.

The intervention evidence relevant to the framework remains encouraging but geographically limited. Culturally adapted cognitive behavioral therapy (CBT) showed large effect sizes for depression and anxiety across nine studies of Arab adults (Kayrouz et al., 2018), while internet-delivered interpersonal therapy showed promise for somatoform symptoms, a finding potentially relevant to the pattern of somatization documented in Saudi primary care (El-Haj-Mohamad et al., 2024). Islamic-integrated CBT studies from Pakistan have reported sustained effects, and an Islamic-based randomized controlled trial (RCT) reported a substantial reduction in anxiety among women (Saged et al., 2022). These findings demonstrate the potential of culturally and religiously adapted approaches but cannot be assumed to establish effectiveness among Saudi women. Critically, no randomized controlled trial identified in the present synthesis tested a culturally adapted psychological intervention for anxiety or depression with Saudi or GCC women as the primary population and clinical outcome measurement. Qualitative adaptation work in Saudi Arabia and Bahrain (Algahtani et al., 2019) and the ongoing Qatar postpartum-depression prevention RCT (Ahmed et al., 2023) illustrate emerging regional efforts.

The principal limitation of BPSS-I is therefore the distance between its theoretical formulation and its empirical validation. The framework is theory-generating and should be tested prospectively in Saudi populations to determine whether its proposed domains and cross-domain pathways are empirically distinguishable, how they interact, and whether they improve explanation, assessment, or intervention beyond existing models. Comparative research could subsequently examine which components remain applicable, which require modification, and which are context-specific across different Gulf, Arab, and Muslim populations. Until such evidence is available, BPSS-I should be interpreted as a Saudi-grounded conceptual framework for hypothesis generation and culturally informed analysis rather than as a validated model with established cross-cultural generalizability.

### 4.5 Limitations

Several limitations should be acknowledged. First, the national surveillance data rely primarily on self-reported measures. As discussed above, self-report may underestimate psychological morbidity in settings where mental-health stigma is substantial and distress may be communicated through somatic rather than explicitly psychological symptoms. Differences in survey instruments, sampling frames, age ranges, and reporting periods across the national sources also preclude direct statistical pooling or simple comparison of prevalence estimates.

Second, an important methodological limitation concerns the reproducibility of the literature synthesis. Although literature identification and abstract-level screening were conducted through a structured search process using predefined eligibility criteria, the final evidence base was not derived from a completed systematic-review process. Full-text retrieval was incomplete, with 275 of the 521 records meeting abstract-level eligibility criteria not retrieved within the available resources and access routes. Consequently, a formal full-text eligibility stage yielding an exhaustive final set of included studies was not undertaken. The narrative synthesis instead relied on purposive thematic selection from the pool of retrievable evidence according to relevance to the predefined review domains. Because final evidence selection therefore depended partly on author judgment regarding thematic and contextual relevance, repetition of the review by other investigators could reasonably result in a different composition of the evidence base. Accordingly, the findings should be interpreted as an interpretive and theory-generating integrative synthesis rather than a strictly reproducible systematic review.

Third, no standardized risk-of-bias or formal methodological-quality assessment was undertaken. Studies of varying designs and methodological quality therefore contributed to the narrative synthesis, although their methodological limitations and contextual relevance were considered during interpretation. In addition, a substantial proportion of the available empirical evidence is cross-sectional, which limits causal and temporal inference. The synthesis may also be affected by publication bias, language bias, incomplete full-text retrieval, and the purposive selection of retrievable evidence. These limitations warrant caution when interpreting apparent associations as explanatory or causal relationships.

Fourth, although the Saudi National Mental Health Survey (SNMHS) remains the most methodologically rigorous national epidemiological investigation available for several outcomes examined in this review, its fieldwork was conducted between 2010 and 2016 and therefore does not directly capture the post–Vision 2030 reform period. The associations discussed between social transformation and women’s mental health should consequently not be interpreted as demonstrating causal effects of the reforms. Longitudinal studies spanning the pre- and post-reform periods are needed to establish whether and how women’s mental-health outcomes have changed over time.

Fifth, most national datasets and many of the reviewed studies do not adequately distinguish between Saudi and non-Saudi women. This limits assessment of how nationality, migration status, cultural background, and socioeconomic circumstances intersect with gender-specific mental-health outcomes and constrains the generalizability of some national estimates to particular population groups.

Sixth, the proposed Islamic Biopsychosocial–Spiritual (BPSS-I) framework is a conceptually derived, theory-generating model and has not yet been empirically validated in Saudi Arabia or the wider GCC. Its present contribution is therefore explanatory and hypothesis-generating: it provides a culturally grounded structure for integrating biological, psychological, social, family-system, and spiritual determinants identified across the reviewed evidence. Prospective empirical studies are required to evaluate its construct validity, explanatory utility, predictive performance, and potential clinical applicability.

Finally, some of the evidence concerning stigma mechanisms, supernatural attributions, family influences, and help-seeking behaviors derives from studies conducted in other GCC settings, including Kuwait, the United Arab Emirates, and Qatar. Although these contexts share certain religious, cultural, and family-structural characteristics with Saudi Arabia, they are not culturally or institutionally interchangeable. Differences in social organization, population composition, legal context, and healthcare systems limit direct transferability. Such evidence was therefore used primarily to contextualize mechanisms for which Saudi-specific research remains limited, and the corresponding interpretations should be regarded as provisional pending further Saudi-specific investigation.

## 5 Conclusion

This article argues that Saudi women bear a disproportionate and largely invisible burden of anxiety and depression, shaped by interlocking biological vulnerabilities, psychological mechanisms, social-structural constraints, and spiritual determinants that standard epidemiological surveillance captures poorly and that Western-derived clinical frameworks address inadequately when applied without cultural adaptation. Four conclusions emerge from the integrated reading of national data and comparative literature.

First, the measured gender gap may not fully capture the underlying burden of anxiety and depression among Saudi women. National figures showing severe anxiety in 9.0% of women versus 6.9% of men, and moderate depression in 0.2% versus 0.1%, should be interpreted cautiously given their reliance on self-reported measures and the potential influence of stigma and somatic expressions of psychological distress. Evidence from Arab primary-care settings reporting substantial levels of somatized psychiatric presentation, together with findings of limited physician agreement in detecting somatized depression in Riyadh, suggests that some psychological morbidity may remain underrecognized in routine surveillance and clinical practice. These findings do not establish the magnitude of any underestimation but indicate an important measurement concern. Addressing this gap would benefit from complementing national self-report surveys with clinician-administered diagnostic assessments, validated Arabic instruments sensitive to somatic presentations of psychological distress, and service-utilization indicators that may help identify otherwise underrecognized mental-health needs.

Second, the treatment gap appears to reflect an interaction of cultural, social, and structural barriers rather than service availability alone. National evidence indicating that approximately 80% of individuals with severe mental disorders receive no care underscores the magnitude of this gap but does not, by itself, establish its underlying causes. Available evidence suggests that family-related stigma and concerns about collective reputation may be particularly relevant to help-seeking among Saudi women, as mental-health disclosure can extend beyond an individual health decision to involve perceived implications for family relationships and social standing. These patterns support broadening intervention strategies beyond individual attitudes to include family and community contexts. Potential approaches include culturally appropriate family-engagement and community-based destigmatization initiatives, the careful integration of Islamic concepts that support health-seeking and psychological care, and confidential or digitally delivered pathways that may reduce perceived disclosure risks. These approaches should be empirically evaluated in Saudi populations to determine their acceptability, effectiveness, and potential contribution to narrowing the gap between willingness to seek help and actual access to mental-healthcare services.

Third, Vision 2030 reforms have substantially altered the structural and economic context of Saudi women’s participation in the labor market, while the available evidence does not yet establish whether these changes have been accompanied by corresponding improvements in psychological wellbeing. Cross-sectional findings concerning distress among working women are consistent with the possibility that expanded occupational opportunities may coexist with pressures arising from multiple work and family roles, but they cannot establish either the direction or causality of this relationship. Whether greater structural and economic participation ultimately translates into mental-health benefits may depend partly on how changes in formal opportunities interact with domestic role expectations, childcare provision, workplace flexibility, and access to occupational mental-health support. These relationships require longitudinal Saudi-specific investigation capable of distinguishing the effects of labor-market participation from broader social, demographic, and economic influences. From a policy perspective, integrating mental-health support into initiatives accompanying women’s expanding workforce participation may therefore complement the broader human-capital and wellbeing objectives associated with Saudi Vision 2030.

Fourth, the Islamic Biopsychosocial–Spiritual (BPSS-I) framework is offered as a culturally coherent organizing heuristic -proposed, not yet validated -for assessment and intervention design. It extends the biological, psychological, and social domains of the original model with a spiritually operationalized fourth domain specific to the Islamic context. Its proposed clinical translations map onto existing evidence in ways that are testable rather than established: internet-delivered culturally adapted CBT could address the somatization-detection failure and the stigma barrier to in-person care simultaneously; Islamic-integrated CBT -drawing on hypothesized correspondences between *tawakkul* and acceptance-based approaches, *sabr* and behavioral activation, and *dhikr* and mindfulness techniques -could provide a therapeutically coherent vocabulary for Saudi women; family-systems integration reflects the relational rather than individual construction of identity in collectivist contexts; and gender-sensitive service models such as the Qatar initiative offer an institutional template. These remain proposals awaiting direct empirical test.

The single most urgent priority is the absence of any randomized controlled trial testing a culturally adapted psychological intervention for anxiety or depression with Saudi or GCC women as the primary population and clinical outcome measurement. Alongside such trials, longitudinal cohort studies tracking mental-health trajectories across the Vision 2030 reform period, clinician-administered epidemiological surveys to complement self-report surveillance, and empirical validation of the BPSS-I framework in Saudi primary-care and occupational settings constitute the research agenda that evidence-based policy for Saudi women’s mental health now requires.

The women whose distress is rendered invisible by somatization, familialism, and diagnostic skew are not absent from the healthcare system -they are present in gastroenterology clinics, orthopedic departments, and general-practice waiting rooms, carrying the somatic language of suffering that their social world permits. Making that suffering visible, and responding with care that is clinically rigorous and culturally coherent, is both a public-health imperative and a prerequisite for the equitable human development that Vision 2030 aspires to.

## Data Availability

The original contributions presented in the study are included in the article/supplementary material, further inquiries can be directed to the corresponding author.
